# Comparation of magnetic resonance hysterosalpingography and hysterosalpingosonography for the assessment of fallopian tubal occlusion of female infertility: A protocol for systematic review and meta-analysis: Retraction

**DOI:** 10.1097/MD.0000000000042736

**Published:** 2025-06-13

The article, “Comparation of magnetic resonance hysterosalpingography and hysterosalpingosonography for the assessment of fallopian tubal occlusion of female infertility: A protocol for systematic review and meta-analysis”,^1^ which appears in Volume 101, Issue 3 of *Medicine*, is being retracted over concerns with the methodology. After publication, concerns were raised over references to the “Begger test,” which does not exist in the literature and is indicative of possible fraud. Upon further investigation, authorship irregularities were also discovered in our submission system. As a result, the Publisher no longer has confidence in the integrity of the content or the authorship.
